# Metabolaid^®^ Combination of Lemon Verbena and Hibiscus Flower Extract Prevents High-Fat Diet-Induced Obesity through AMP-Activated Protein Kinase Activation

**DOI:** 10.3390/nu10091204

**Published:** 2018-09-01

**Authors:** Young-Sil Lee, Won-Kyung Yang, Hwa Yeon Kim, Bokkee Min, Nuria Caturla, Jonathan Jones, Yang-Chun Park, Young-Cheol Lee, Seung-Hyung Kim

**Affiliations:** 1 Herbal Medicine Research Division, Korea Institute of Oriental Medicine, 1672 Yuseong-daero, Yuseong-gu, Dajeon 34054, Korea; rheeys04@kiom.re.kr; 2 Institute of Traditional Medicine and Bioscience, Daejeon University, 62 Daehak-ro, Dong-gu, Daejeon 34520, Korea; ysks1220@dju.kr; 3 Novarex, 94, Gangni 1-gil, Ochang-eup, Cheongwon-gu, Cheongju-si, Chungcheongbuk-do 368-885, Korea; khay228@novarex.co.kr (H.Y.K.); bokkee@novarex.co.kr (B.M.); 4 Monteloeder, S.L., Miguel Servet 16, Nave 17, 03203 Elche, Spain; nuriacaturla@monteloeder.com (N.C.); jonathanjones@monteloeder.com (J.J.); 5 Division of Respiratory Systems, Department of Internal Medicine, College of Korean Medicine, Daejeon University, Daejeon 34520, Korea; omdpyc@dju.kr; 6 Department of Herbology, College of Korean Medicine, Sangji University, Wonju 220-702, Korea; lyc072@sangji.ac.kr

**Keywords:** lemon verbena, hibiscus, obesity, hyperlipidemia, adipogenesis, thermogenesis, AMP-activated kinase

## Abstract

Lemon verbena (*Lippia citriodora*) has been used as a food spice, cosmetic, and in traditional medicine formulations to treat asthma and diabetes in South America and Southern Europe. Hibiscus flower (*Hibiscus sabdariffa* L.) is used in traditional Chinese medicine in the form of a tea to treat hypertension and inflammation. In the present study, we examined the synergistic effects of a formula of Metabolaid^®^ (MetA), a combination of lemon verbena and hibiscus-flower extracts, on obesity and its complications in high-fat-diet (HFD)-induced obese mice. The results showed that MetA decreased body weight, white adipose tissue (WAT), and liver weight. Additionally, serum and hepatic lipid profiles, glucose levels, glucose tolerance, and cold-induced thermogenesis were significantly improved. Appetite-regulating hormones adiponectin and leptin were significantly increased and decreased, respectively, while the inflammatory-related factors tumor necrosis factor (TNF)-α and interleukin (IL)-6 were downregulated by MetA. Adipogenesis-activating gene expression was decreased, while increased thermogenesis-inducing genes were upregulated in the WAT, correlating with increased phosphorylation of AMPK and fatty-acid oxidation in the liver. Taken together, these results suggest that MetA decreased obesity and its complications in HFD mice. Therefore, this formula may be a candidate for the prevention and treatment of obesity and its complications.

## 1. Introduction

Obesity characterized by excessive fat mass and dysregulation of lipid metabolism is a significant public-health problem worldwide, contributing to the development of metabolic diseases such as type 2 diabetes, hyperlipidemia, hypertension, and cardiovascular disease, which increase morbidity and mortality [1,2]. Currently, pharmacological agents such as orlistat, lorcaserin, and phentermine/topiramate have been used to treat obesity. However, these medications are associated with various adverse effects such as steatorrhea, insomnia, vomiting, headache, stomachache, and gastrointestinal disturbance [3]. Therefore, natural products have emerged as an attractive source for the prevention and treatment of obesity owing to their efficacy and fewer side effects than those of synthetic agents [4]. Recently, an increasing body of evidence of the beneficial effects of botanical ingredients such as *Garcinia cambogia* (GC), bitter orange, and green coffee, which have shown promising results in preventing and treating obesity and its health complications [5].

Lemon verbena (*Lippia citriodora*) has been widely used as a food spice, cosmetic, and in traditional medicine formulations to treat asthma, colds, fevers, flatulence, colic, diarrhea, and diabetes in South America and Southern Europe [6,7]. Pharmacological studies of lemon verbena have reported it to have important antioxidant and anti-inflammatory effects [8], even on the adipose tissue, where it was shown to decrease lipid accumulation and hyperlipidemia [9]. On the other hand, the hibiscus flower (*Hibiscus sabdariffa* L.) is regularly used in traditional Chinese medicine in the form of a tea to treat hypertension, oxidative stress, and inflammation, as well as kidney and urinary bladder stones [10,11]. According to various reports, the extracts and polyphenols of hibiscus inhibit lipid accumulation in adipocytes and reduce pancreatic amylase, as well as cholesterol and triglyceride levels in animal models [12,13,14,15,16,17]. Collectively, these observations indicate that lemon verbena and hibiscus can restore dysregulated glucose and lipid metabolism, suggesting a possible application in treating obesity and its consequences, including fatty liver, hyperlipidemia, and insulin resistance. Furthermore, it has been reported that combining various herbal extracts may synergistically increase their effectiveness, as well as decrease the toxic effects of certain drugs [18]. The synergistic effects of lemon verbena and hibiscus on the adipose tissue have not been previously examined. Thus, in the present study, we aimed to elucidate whether the combination of lemon verbena and hibiscus could synergistically improve the metabolism of obese mice fed a high-fat diet (HFD).

## 2. Materials and Methods

### 2.1. Plant Material Extraction and Extract Combination Preparation

Lemon verbena leaf extract (*L. citriodora*, LV), hibiscus flower (*H. sabdariffa* L., HS) extract, and the combination (Metabolaid^®^, MetA) were provided by Monteloeder S. L (Alicante, Spain). GC extract was provided by Novarx (Cheongju-si, Korea), and the LV and HS were prepared by hydroalcoholic extraction, centrifuged, concentrated under reduced pressure using a rotary evaporator, and then filtered. The combined product, MetA, was prepared by mixing LV and HS extracts at a weight ratio (*w*/*w*) of 65:35. The powder form of the extract was analyzed for microorganisms, including bacteria, yeast, and mold, by a certified laboratory of analysis. The certificate of analysis is available (in Spanish). The result of coliform bacteria test was negative in MetA (mixture). The powder form of the extract was prepared in bulk, and stored in controlled temperature and humidity. Stability tests have been performed with the powder form, in both normal and accelerated conditions. The extract has a shelf-life of at least 12 months.

### 2.2. HFD Mice and Administration of LV, HS, and MetA

Eight-week-old male C57BL/6J mice were purchased from The Jackson Laboratory (Bar Harbor, ME, USA) and housed in a temperature-controlled room, at a stable temperature of 21 ± 2 °C and humidity of 50 ± 5% under a 12-h light–dark cycle. The mice were fed a commercial diet and water ad libitum for 1 week. Mice were then fed an HFD (Rodent Diet D12492, Research Diet, New Brunswick, NJ, USA) consisting of 60% fat, 20% protein, and 20% carbohydrate to induce obesity. Normal-control mice were fed+ a standard diet (AIN-76A, Research Diet, New Brunswick, NJ, USA) consisting of 11.5% fat, 20.8% protein, and 67.7% carbohydrate. The animal feeds were purchased from Research Diets Inc. (New Brunswick, NJ, USA). The mice were randomly divided into the following groups of 10 mice each, and were fed as indicated: normal diet (ND) and HFD, while three groups were each fed the HFD plus the LV extract (HFD + LV), HS extract (HFD + HS), and the MetA complex (HFD + LV + HB) for 8 weeks. LV, HB (100 mg/kg each), and the MetA (50 and 100 mg/kg) samples were condensed to the dose of 24.5 mg/mL, 10 mg/mL, 10 mg/mL, 5 mg/mL and 10 mg/mL, respectively, in 0.5% CMC solution and were given orally to the mice once daily with volume based on body weight (g) at 09:30–10:30 for 8 weeks. GC (245 mg/kg) was used as the positive control treatment since its effects are well known, and it is available to the public as an antiobesity agent. The normal- and HFD-control mice were treated with the vehicle (normal saline) only. Body weight and food intake were monitored once weekly. Obesity is generally defined as being 20% or more over the ideal body weight. After 1 week of feeding, the body weight of the HFD group was approximately 20% higher than that of the normal-control group. All animal procedures were performed according to the Guide for the Care and Use of Laboratory Animals of the National Institutes of Health and were approved by the Institutional Animal Care and Use Committee of Daejeon University in Daejeon, Korea (Approval No. DJUARB2016-032). Body-weight gain and the food intake were measured at the same time and the same day of the week during the 9-week experimental period. The average body-weight gain and food intake were calculated daily and recorded. Food-efficiency ratio was calculated as follows: food efficiency ratio (%) = (body weight gain (g/day)/food intake (g/day)) × 100.

### 2.3. Tissue Weight and Histological Analysis

At the end of the experimental period, the mice were fasted for 15 h and then euthanized. After blood collection, the abdominal subcutaneous fat (abdominal subWAT), epididymal WAT (EWAT), retroperitoneal WAT (RWAT), and intestinal WAT, and the liver, kidney, and spleen were removed and immediately weighed. For adipocyte staining, the EWAT and liver were fixed in 10% neutral formalin solution for 24 h and then embedded in paraffin. All the tissue samples were cut into 6 μm-thick sections and stained with hematoxylin and eosin (H and E) or Oil Red O. To measure the size of the adipocytes, the area comprising 20 adipocytes in stained sections was measured using a light microscope (Olympus BX51, Olympus Optical Co., Tokyo, Japan) using an image-analysis program (Image-Pro Plus 5.0, Media Cybernetics, Silver Spring, MD, USA). Histological analysis of the collected tissue samples was performed.

### 2.4. Serum Assay of Biochemical Parameters 

At the end of the 9-week experimental period, the mice were fasted for 15 h prior to euthanasia. Blood samples were centrifuged at 1100 g for 15 min at 4 °C. The separated serum samples were stored at −70 °C. The serum levels of triglyceride (TG), total cholesterol (T-CHO), high-density lipoprotein cholesterol (HDL-CHO), low-density lipoprotein cholesterol (LDL-CHO), glucose, alanine aminotransferase (ALT), aspartate aminotransferase (AST), creatinine, nonesterified fatty acid (NEFA), and TG level in the liver tissue were analyzed using an automatic biochemical analyzer (Hitachi-7020, Hitachi Medical, Tokyo, Japan). The serum concentrations of leptin, adiponectin, and insulin growth factor (IGF)-1 level were assayed using a mouse enzyme-linked immunosorbent assay (ELISA) kits (R and D Systems, Minneapolis, MN, USA).

### 2.5. Rectal-Temperature Measurement in Cold Response 

At the end of 8 weeks on the HFD (at 18-week-old), the rectal temperatures of the mice were measured in the cold chamber at an ambient temperature of 10 °C. The animals were sedated and restrained for <30 s during the measurement. Next, LV, HS (100 mg/kg each), MetA (50 and 100 mg/kg), and GC (245 mg/kg) were orally administered to respective groups, and their rectal temperatures were measured at regular intervals (30, 60, 90, 120, and 180 min) after sample administration. A Thermalert model TH-8 temperature monitor (Physitemp, Clifton, NJ, USA) was used with the probe placed 2.5 cm deep in the rectum.

### 2.6. Intraperitoneal Glucose Tolerance Test (IGTT)

All the mice were fasted for 12 h overnight at the end of the preventive experiment after 8 weeks on the HFD (at 18 weeks old). For the IGTT, blood samples were collected from the tail vein for determination of baseline glucose values (0 min). Next, intraperitoneal injections of glucose (1 g/kg body weight) were administered to all the mice after 30 min, and blood-glucose levels were measured at regular intervals (30, 60, and 120 min) after the injection of glucose. The blood-glucose level was measured using a SureStep Plus Glucometer (LifeScan, Milpitas, CA, USA).

### 2.7. Quantitative Real-Time Polymerase Chain Reaction (qPCR)

The abdominal subcutaneous WAT and EWAT were homogenized, and the total RNA was isolated using TRI reagent (Sigma-Aldrich, St. Louis, MO, USA) and digested with DNase I (Life Technologies, Grand Island, NY, USA) to remove the chromosomal DNA according to the manufacturer’s protocol. The primers used for the qPCR are listed in Table 1.

### 2.8. Western Blotting Analysis

Following blood collection, the mice were euthanized with diethyl ether, and the liver tissues were immediately removed and placed in liquid nitrogen for storage at −70 °C. Protein extracts were prepared using a protein-extraction kit (Intron Biotechnology Inc., Seoul, Korea). Lysates (50 μg) were electroblotted onto a nitrocellulose membrane following separation on 8% sodium dodecyl sulfate (SDS) polyacrylamide gel electrophoresis. Blotted membranes were incubated for 1 h with blocking solution (trisbuffered saline/Tween 20, TBST) containing 5% skimmed milk (*w*/*v*), followed by incubation overnight at 4 °C with a 1:1000 dilution of primary antibodies against AMP-activated protein kinase (AMPK), phosphorylated AMPK (p-AMPK), ACC, or p-ACC (Cell Signaling Technology, Beverley, MA, USA). Membranes were washed 4 times with 0.1% TBST and incubated with a 1:3000 dilution of horseradish peroxidase-conjugated goat antirabbit or donkey antirabbit IgG secondary antibody (Santa Cruz Biotechnology, Santa Cruz, CA, USA) for 1 h at room temperature. Membranes were washed 4 times in TBST and then developed using electrochemiluminescence (Amersham, GE Healthcare, Uppsala, Sweden). The ImageJ 1.49 software (http://rsb.info.nih.gov/ij/download.html; National Institutes of Health (NIH)) was used for the quantification of the results of the western blotting.

### 2.9. High-Performance Liquid Chromatography (HPLC) Analysis of MetA

MetA was analyzed using an HPLC system (Waters Alliance 2695 system, Waters Co., Milford, MA, USA) comprising a 2996-photodiode array detector (PDA) equipped with a BDS hypersil C_18_ column (250 × 4.6 mm, 5 μm particle size; Thermo Fisher, Waltham, MA, USA). The mobile phase was composed of 10% (*v*/*v*) formic acid aqueous solution (A) and methanol (B). The elution conditions were as follows: 0–35 min with 95% A and 5% B, 35–40 min with 60% A and 40% B, 40–45 min with 95% A and 5% B, and at 45 min back to initiation conditions with 95% A and 5% B. The flow rate was 1.0 mL/min at a temperature of 25 °C with an injection volume of 20 μL. For the maker compounds, the peak area was determined at a wavelength of 330 for verbascoside and isoverbascoside and 520 nm for anthocyanins, and the compounds were identified by comparing the retention times and ultraviolet (UV) spectra of the peaks of the HPLC/PDA chromatograms to those of commercially available standards. MetA and standard compounds were prepared with 50% (*v*/*v*) methanol solution.

### 2.10. Statistical Analysis

Differences between groups were assessed using an analysis of variance (ANOVA) followed by Tukey’s multiple-range test. All data are presented as the means ± standard error of the mean (SEM). Differences were considered significant at a *p* < 0.05.

## 3. Results

### 3.1. HPLC Analysis of MetA

**Figure 1:**
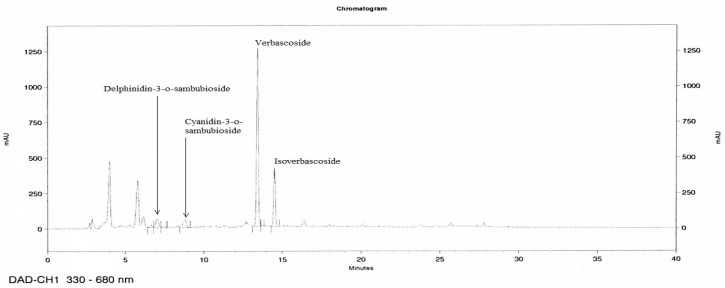
Representative high-performance liquid chromatography (HPLC) chromatograms of the Metabolaid^®^ (MetA). HPLC chromatograms of two extract mixtures at 320 and 520 nm. Delphinidin-3-o-sambubioside, cyanidin-3-o-sambubioside, verbascoside, and isoverbascoside appeared at retention times of 7.1, 8.8, 13.4, and 14.5 min, respectively.

HPLC chromatograms of MetA show the four compounds, delphinidin-3-o-sambubioside, cyanidin-3-o-sambubioside, verbascoside, and isoverbascoside identified as the main active compounds (Figure 1). Verbascoside and isoverbacoside contents were 15.0–18.0% and 1–3%, respectively.

### 3.2. Effect of LV, HS, and the MetA on Body Weight and Food Intake of HFD-Induced Obese Mice

**Figure 2:**
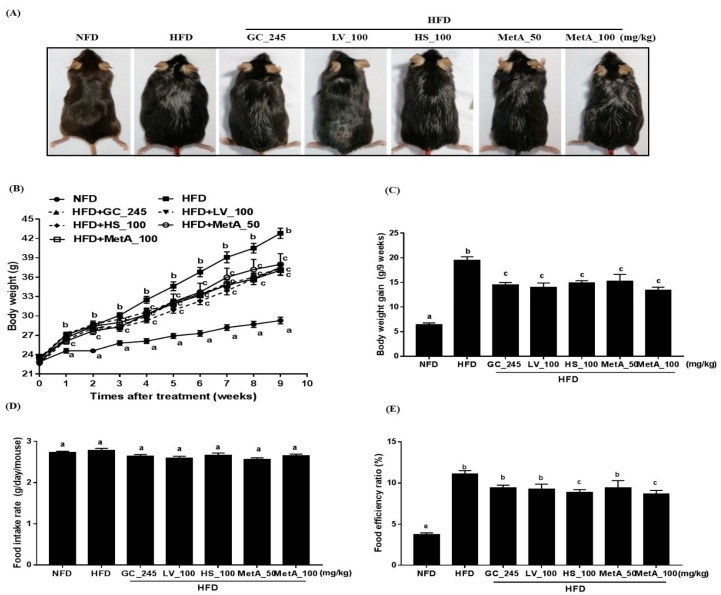
Effect of lemon verbena (LV), Hibiscus flower (HS), and their combination (MetA) on body weight and food intake in high-fat-diet (HFD)-induced obese mice. Changes in body size (**A**), body weight (**B**), body weight gain (**C**), food intake (**D**), and food efficiency ratio (FER, %, **E**) of normal diet group (NFD) and high fat diet-induced obese(DIO) mice after 9 weeks of 60% HFD feeding. HFD, 60% high-fat-diet control group; HFD + GC_245, HFD contains *Garcinia cambogia* extract (245 mg/kg)-fed group; HFD + LV_100, HFD contains lemon verbena extract (100 mg/kg)-fed group; HFD + HS_100, HFD contains *Hibiscus* flower extract (100 mg/kg)-fed group; HFD + MetA_50, HFD contains the MetA-mixed extract of lemon verbena plus *Hibiscus* flower (50 mg/kg)-fed group; HFD + MetA_100, HFD contains the MetA-mixed extract of lemon verbena plus *Hibiscus* flower (100 mg/kg)-fed group. Values are means ± SEM (*n* = 10). ^a–c^ Values within arrow with different letters are significantly different from each other at *p* < 0.05 as determined using Turkey’s multiple-range test.

The body size, weight, and weight gain were significantly lower in the NFD-, GC-, LV-, HS-, and MetA-treated groups than they were in the HFD group (Figure 2A–C). Although there was no significant difference in the food-intake rate among the groups, food-efficiency ratio (FER) was significantly lower in the all treated groups than it was in the HFD group, and a more significantly pronounced effect was observed in the HS and MetA 100 groups (Figure 2D,E).

### 3.3. Effect of LV, HS, and the MetA on Adipose Tissue Weight and Morphology of HFD-Induced Obese Mice

**Figure 3:**
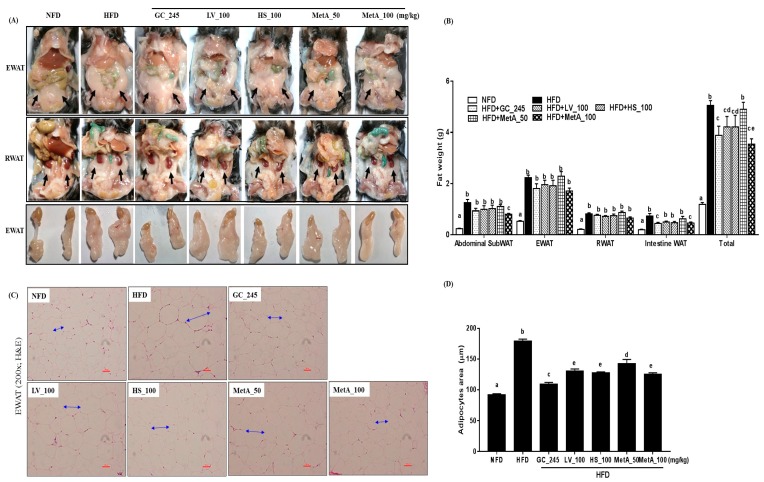
Effect of LV, HS, and MetA on WAT weight and morphology in HFD-induced obese mice. Representative images (**A**), final WAT weight (**B**), hematoxylin and eosin (H and E) staining of WAT (**C**), and adipocytes area (**D**) in NFD and DIO mice after 9 weeks of 60% HFD feeding. Images were captured under a light microscope at ×100 magnification. Abdominal subWAT, abdominal subcutaneous fat; EWAT, epididymal white adipose tissue; RWAT, retroperitoneal white adipose tissue; Total, abdominal subWAT + EWAT + RWAT + intestine adipose tissue. HFD, 60% high-fat-diet control group; HFD + GC_245, HFD contains *Garcinia cambogia* extract (245 mg/kg)-fed group; HFD + LV_100, HFD contains lemon verbena extract (100 mg/kg)-fed group; HFD + HS_100, HFD contains *Hibiscus* flower extract (100 mg/kg)-fed group; HFD + MetA_50, HFD contains the MetA-mixed extract of lemon verbena plus *Hibiscus* flower (50 mg/kg)-fed group; HFD + MetA_100, HFD contains the MetA-mixed extract of lemon verbena plus *Hibiscus* flower (100 mg/kg)-fed group. Values are expressed as means ± SEM (*n* = 10). ^a–e^ Values within arrow with different letters are significantly different from each other at *p* < 0.05 as determined using Turkey’s multiple-range test.

The EWAT and RWAT were significantly lower in the NFD, GC, LV, HS, MetA50, and MetA100 groups than they were in the HFD group (Figure 3A). All WATs weights in the NFD group were significantly lower than those of the HFD group were. Abdominal SubWAT and intestinal WAT in the MetA100 group was significantly decreased compared with that of the HFD group but not the GC, LV, HS, and MetA50 groups. EWAT and RWAT weights were comparable between the HFD and all treated groups. Regarding total WAT, the weights were significantly reduced in the GC, LV, HS, and MetA100 groups, and, interestingly, the reduction in the MetA100 group was significantly higher than that of the LV and HS alone (Figure 3B), indicating a synergistic effect between the two plant extracts. Histologically, the lipid-droplet value was lower in the NFD, GC, LV, HS, and MetA 100 groups than compared with the HFD group using H and E and oil-red O staining (Figure 3C). In addition, the adipocytes size was significantly larger in the HFD mice than it was in the mice administered GC, LV, HS, and MetA (Figure 3D).

### 3.4. Effect of LV, HS, and MetA on Expression of Adipogenic Regulator Genes in HFD-Induced Obese Mice

**Figure 4:**
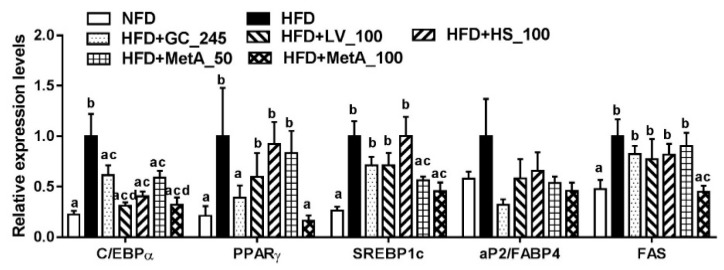
Effect of LV, HS, and MetA on expression of adipogenic regulator genes in HFD-induced obese mice. The mRNA expression levels of adipogenic regulator genes were analyzed using quantitative real-time polymerase chain reaction (qPCR). HFD, 60% high-fat-diet control group; HFD + GC_245, HFD contains *Garcinia cambogia* extract (245 mg/kg)-fed group; HFD + LV_100, HFD contains lemon verbena extract (100 mg/kg)-fed group; HFD + HS_100, HFD contains *Hibiscus* flower extract (100 mg/kg)-fed group; HFD + MetA_50, HFD contains the MetA-mixed extract of lemon verbena plus *Hibiscus* flower (50 mg/kg)-fed group; HFD + MetA_100, HFD contains the MetA-mixed extract of lemon verbena plus *Hibiscus* flower (100 mg/kg)-fed group. Values are expressed as means ± SEM (*n* = 10). ^a–d^ Values within arrow with different letters are significantly different from each other at *p* < 0.05 as determined using Turkey’s multiple-range test.

The CCAAT/enhancer-binding protein (C/EBPα) mRNA expression levels in all the groups were significantly decreased compared to that in the HFD group. Peroxisome proliferator-activated receptor γ (PPARγ) mRNA expression levels were decreased in the NFD, GC, and MetA100 groups, and sterol regulatory element-binding transcription factor 1c (SREBP1c) mRNA expression levels were decreased in the NFD and the two MetA groups compared with the HFD group. However, aP2/FABP4 mRNA expression levels did not differ among all groups. Fatty acid synthase (FAS) mRNA expression levels were decreased in the MetA100 group compared with the HFD group, and these levels were similar to that of the NFD group (Figure 4).

### 3.5. Effect of LV, HS, and MetA on Energy Expenditure and Thermogenesis-Related Gene Expression in HFD-Induced Obese Mice

**Figure 5:**
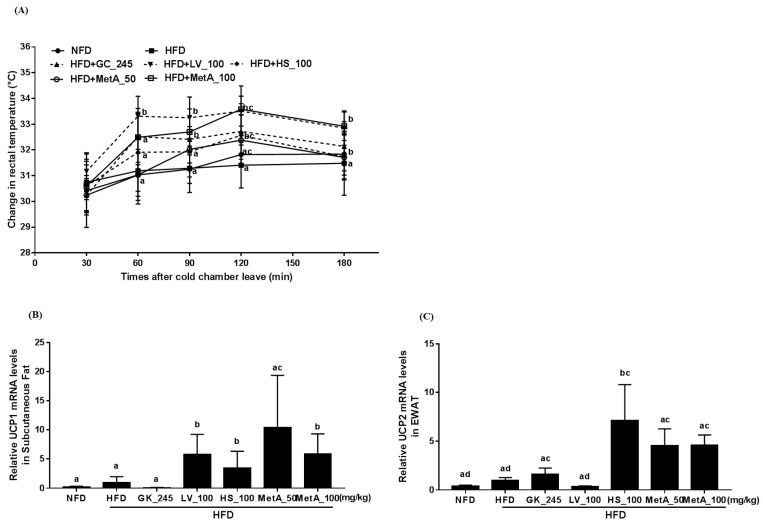
Effect of LV, HS, and MetA on energy expenditure and thermogenesis-related gene expression in HFD-induced obese mice. Rectal temperature (**A**), UCP-1 mRNA expression in subcutaneous fat (**B**), and UCP-2 mRNA expression in EWAT (**C**) of NFD and DIO mice after 9 weeks of 60% HFD feeding. Rectal temperature was measured at regular intervals (30, 60, 90, 120, and 180 min) after sample administration when the mice were exposed to 10 °C ambient temperature in a cold room at 8 weeks on HFD (at 18 weeks of age). Gene-expression levels were analyzed using qPCR. HFD, 60% high-fat-diet control group; HFD + GC_245, HFD contains *Garcinia cambogia* extract (245 mg/kg)-fed group; HFD + LV_100, HFD contains lemon verbena extract (100 mg/kg)-fed group; HFD + HS_100, HFD contains *Hibiscus* flower extract (100 mg/kg)-fed group; HFD + MetA_50, HFD contains the MetA-mixed extract of lemon verbena plus *Hibiscus* flower (50 mg/kg)-fed group; HFD + MetA_100, HFD contains the MetA-mixed extract of lemon verbena plus *Hibiscus* flower (100 mg/kg)-fed group. Values are expressed as means ± SEM (*n* = 10). ^a–d^ Values within arrow with different letters are significantly different from each other at *p* < 0.05 as determined by Turkey’s multiple-range test.

Cold-stimulated adaptive thermogenesis was assessed to determine its association with energy expenditure and contribution to the reduction of body weight gain. As shown in Figure 5A, the rectal temperature under cold exposure was increased in the LV at all time points except 30 min and the MetA100 group at 60, 90, 120, 180 min compared with the HFD group (Figure 5A). In addition, thermogenesis-related gene UCP1 mRNA expression levels were significantly increased in the WAT of the LV, HS, and Met 100 groups (Figure 5B) and UCP2 mRNA expression levels increased in the WAT of the GC, HS, and two MetA groups compared with the HFD group (Figure 5C).

### 3.6. Effect of LV, HS, and MetA on Biochemical Parameters of HFD-Induced Obese Mice

Serum TG, T-CHO, and LDL-CHO levels were significantly decreased in the NFD group compared with that of the HFD group. Serum TG levels were significantly decreased in the LV group compared with the HFD group. Serum NEFA levels were significantly decreased in the LV, HS, and MetA100 groups compared with the HFD groups. Serum T-CHO levels in the GC and MetA100 groups were considerably reduced compared with that in the HFD, and the effect was most significant in the MetA100 group. Serum LDL-CHO levels were reduced in the GC and two MetA groups, whereas HDL-CHO levels were increased in the HS and MetA50 group compared with the HFD group. Serum ALT levels were decreased in all treated groups compared with the HFD group. However, there was no significant difference in the serum AST and creatinine levels among the treated groups. TG levels in the liver were significantly lowered in the GC and MetA100 groups than those of the HFD group were (Table 2).

### 3.7. Effect of LV, HS, and MetA on Systemic Glucose Tolerance in HFD-Induced Obese Mice

**Figure 6:**
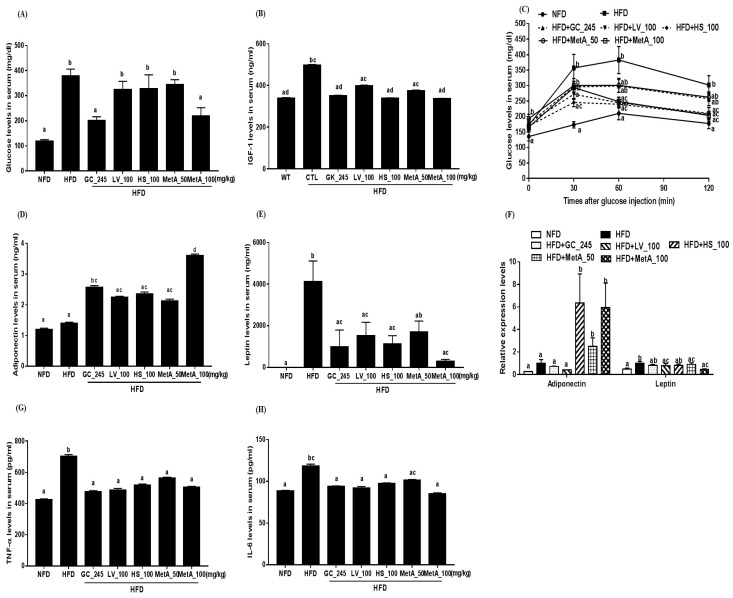
Effect of LV, HS, and MetA on systemic glucose tolerance in HFD-induced obese mice. Serum glucose levels (**A**), serum IGF-1 levels (**B**), intraperitoneal glucose tolerance test (**C**), serum adiponectin levels (**D**), serum leptin levels (**E**), adiponectin and leptin mRNA expression in EWAT (**F**), serum TNF-α levels (**G**), and serum IL-6 levels (**H**). Adipokines were quantified using enzyme-linked immunosorbent assay (ELISA) and an intraperitoneal glucose tolerance test (IGTT) was performed at 8 weeks on HFD (at 18 weeks of age). HFD, 60% high fat diet control group; HFD + GC_245, HFD contains *Garcinia cambogia* extract (245 mg/kg)-fed group; HFD + LV_100, HFD contains lemon verbena extract (100 mg/kg)-fed group; HFD + HS_100, HFD contains *Hibiscus* flower extract (100 mg/kg)-fed group; HFD + MetA_50, HFD contains the MetA-mixed extract of lemon verbena plus *Hibiscus* flower (50 mg/kg)-fed group; HFD + MetA_100, HFD contains the MetA-mixed extract of lemon verbena plus *Hibiscus* flower (100 mg/kg)-fed group. Values are expressed as means ± SEM (*n* = 10). ^a–d^ Values within arrow with different letters are significantly different from each other at *p* < 0.05 as determined using Turkey’s multiple-range test.

The GTT was performed to assess if reducing body weight improved glucose metabolism. The result showed that serum glucose levels were markedly reduced in the NFD, GC, and MetA100 groups compared with that in the HFD group (Figure 6A). Serum IGF-1 levels were decreased in the GC, HS, and MetA100 groups compared with that in the HFD, and these levels were similar to those of the NFD group (Figure 6B). In addition, serum glucose levels did not increase in the LV group at 60 min; the HS group at 30, 60, and 120 min; and the MetA100 group at 60 and 120 min after glucose injection in the IGTT (Figure 6C). Serum adiponectin levels were significantly increased in the GC and MetA100 groups compared with the HFD group, and the MetA100 group presented the highest expression levels of adiponectin, while the serum leptin levels were decreased in the NFD and GC, HS, LV, and MetA100 groups, but not the MetA50 group (Figure 6D,E). Adiponectin mRNA expression levels were significantly increased in the HS and two MetA groups, whereas the leptin mRNA expression levels were decreased in the NFD, LV, and two MetA groups compared with the HFD group (Figure 6F). The serum TNF-α and IL-6 levels were decreased in the NFD, GC, LV, HS, and MetA groups compared with the HFD group, although the IL-6 levels did not differ in the MetA50 group (Figure 6G,H).

### 3.8. Effect of LV, HS, and the MetA on Liver Weight and Hepatic Lipid Accumulation 

**Figure 7:**
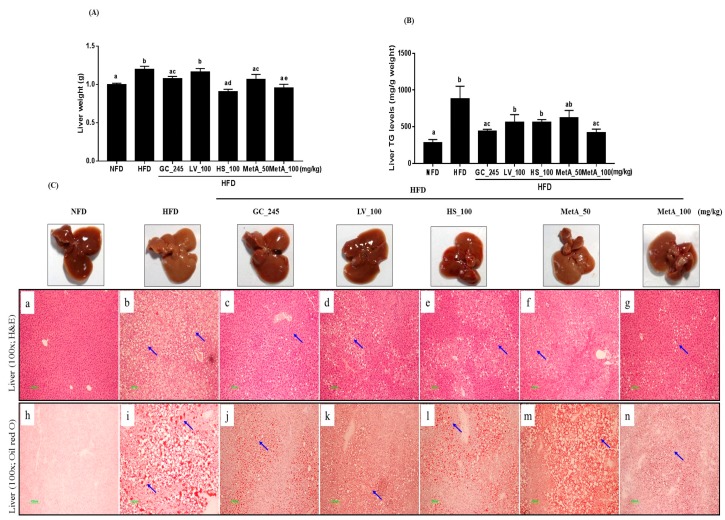
Effects of LV, HS, and MetA on liver weight and hepatic lipid accumulation in HFD-induced obese mice. (**A**) Liver, (**B**) liver TG levels, and (**C**) liver H and E and oil-red O staining. HFD, 60% high-fat-diet control group; HFD + GC_245, HFD contains *Garcinia cambogia* extract (245 mg/kg)-fed group; HFD + LV_100, HFD contains lemon verbena extract (100 mg/kg)-fed group; HFD + HS_100, HFD contains *Hibiscus* flower extract (100 mg/kg)-fed group; HFD + MetA_50, HFD contains the MetA-mixed extract of lemon verbena plus *Hibiscus* flower (50 mg/kg)-fed group; HFD + MetA_100, HFD contains the MetA-mixed extract of lemon verbena plus *Hibiscus* flower (100 mg/kg)-fed group. Values are expressed as means ± SEM (*n* = 10). ^a–d^ Values within arrow with different letters are significantly different from each other at *p* < 0.05 as determined using Turkey’s multiple-range test.

The liver weight was reduced in the NFD, GC, HS, and two MetA groups compared with that of the HFD group, and the largest effect was observed in the MetA100 group (Figure 7A). Liver TG levels were significantly lower in the GC and MetA100 groups than those in the HFD group (Figure 7B). The liver tissues in the all treated groups presented a more normal morphology than that of the pale and enlarged liver observed in the HFD group. Histologically, the lipid-droplet value was lower in the NFD, GC, LV, HS, and MetA 100 groups than compared with the HFD group using H and E and oil-red O staining. In addition, the lipid droplet was significantly larger in the HFD mice than it was in the mice administered GC, LV, HS, and MetA (Figure 7C).

### 3.9. Effects of LV, HS, and the MetA on Lipid Metabolism-Related Genes and Protein Expression in the Liver

**Figure 8:**
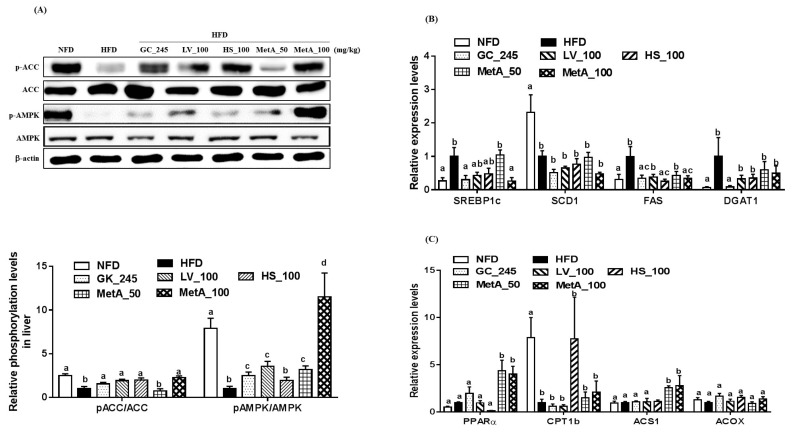
Effects of LV, HS, and MetA on ACC and AMP-activated kinase (AMPK) phosphorylation in liver tissue of obese mice fed HFD. Quantification of proteins in immunoblot (**A**). A representative blot is shown (A, upper), Densitometric analyses of p-ACC and ACC, and p-AMPK and AMPK (A, lower) in liver. Relative signal strength of AMPK, p-AMPK, ACC, p-ACC, and internal control (β-actin) was quantified for each band and the relative expression levels of quantification of proteins were calculated as a ratio to HFD-HFD gene expression. mRNA expression levels of lipogenesis (**B**) and fatty acid oxidation (**C**)-related genes were analyzed using qPCR. HFD, 60% high-fat-diet control group; HFD + GC_245, HFD contains *Garcinia cambogia* extract (245 mg/kg)-fed group; HFD + LV_100, HFD contains lemon verbena extract (100 mg/kg)-fed group; HFD + HS_100, HFD contains *Hibiscus* flower extract (100 mg/kg)-fed group; HFD + MetA_50, HFD contains the MetA-mixed extract of lemon verbena plus *Hibiscus* flower (50 mg/kg)-fed group; HFD + MetA_100, HFD contains the MetA-mixed extract of lemon verbena plus *Hibiscus* flower (100 mg/kg)-fed group. Values are expressed as means ± SEM (*n* = 10). ^a–d^ Values within arrow with different letters are significantly different from each other at *p* < 0.05 as determined using Turkey’s multiple-range test.

The p-ACC levels were markedly increased in the NFD, LV, HS, and MetA100 groups, but not the MetA50 group compared with the HFD group. The p-AMPK levels were increased compared to that of the HFD in all groups except the HS, and the values were most relevant in the MetA100 group where the p-AMPK were even higher than that of the NFD group (Figure 8A). SREBP1c mRNA expression levels significantly decreased in the NFD, GC, and MetA100 groups. FAS mRNA expression levels significantly decreased in the NFD, HS, and MetA100 groups. Stearoly-CoA desaturase1 (SCD1) and diglyceride acyltransferase 1(DGAT1) mRNA expression levels did not differ among treated groups (Figure 8B). PPARα and acetyl-coenzyme A synthase 1 (ACS1) mRNA expression levels significantly increased in the MetA groups, but carnitine palmityltransferase1 (CPT1) and acyl-CoA Oxidase (ACOX) mRNA expression levels did not differ among all groups (Figure 8C).

## 4. Discussion

In the present study, we investigated the effects of LV, HS, and their combination (MetA) on obesity and its complications. We discovered that in mice fed an HFD, MetA, LV, and HS reduced body-weight gain and total adipose-tissue weight, and smaller adipocyte cell sizes were detected. MetA was more effective on WAT weight reduction than LV or HS alone was at the same daily dose. Consistent with reduced fat accumulation and adipocyte size, MetA increased the serum adiponectin levels and mRNA levels, whereas it decreased the serum leptin levels and mRNA levels. In addition, MetA significantly decreased adipogenesis-related genes, CEBP/α, PPARγ, and SREBP-1c but only MetA 100 was able to reduce their target gene FAS mRNA expression levels in WAT. Adipogenic transcription factors such as CEBP/α and PPARγ and their target genes, aP2/FABP4 and FAS, and a complex network of these factors led to lipid accumulation within cells and involved in the development of obesity [19]. These results indicate that MetA, as well as LV and HS, may exert an enhanced antiobesity effect, which may be accompanied by the reduced expression of adipogenesis-related genes, and their combination was more effective than either agent alone. Although there have been reports of the antiobesity effects of LV and HS, this study is the first to demonstrate that the synergistic effects of the combination of these two extracts on obese mice. Furthermore, interestingly GC and MetA exhibited similar effects, and in certain cases performed better, at a much lower daily dose (100 vs. 245 mg/kg of GC).

Obesity is caused by excessive energy intake relative to energy expenditure. In the present study, MetA reduced the food-efficiency ratio without changing the food-intake rate, implying that antiobesity effect by MetA were associated with reduced WAT weight independent of food intake. Thus, we determined that the MetA might increase energy expenditure by increased thermogenesis. Thermogenesis is defined as the energy that is dissipated in the form of heat in response to environmental temperature and diet, which may result in weight loss and obesity prevention [20,21]. Recently, it has been reported that white adipocytes in subcutaneous WAT can acquire a brownlike adipocyte morphology, known as brite/beige adipocytes, which exhibits thermogenic properties [22]. An increase in brite/beige adipocytes in the WAT is linked to the prevention of diet-induced obesity with increased energy expenditure [23]. As UCP1, a key regulator of thermogenesis, is mainly expressed in BAT and brite/beige adipocytes, its increased expression in WAT could be considered as an adequate target for the prevention and treatment of obesity [24]. In addition to UCP1, UCP2 is involved in thermogenesis associated with energy expenditure in WAT [25]. UCP3 is also involved in energy expenditure by decreasing radical oxygen species production and promoting fatty-acid oxidation in muscle, although its function is still not clearly understood [26]. Interestingly, MetA and LV increased the rectal temperature of animals exposed to cold, indicating that MetA may increase thermogenesis and energy expenditure, which contribute to antiobesity effects. Furthermore, MetA, as well as LV and HS, significantly increased UCP1 and UCP2 mRNA expression levels in WAT. Therefore, the increase of energy expenditure by MetA, associated with increased UCP1 and UCP2 mRNA expression levels, can contribute to the browning of WAT, which promotes thermogenesis and energy expenditure. However, further studies are necessary to elucidate this process.

Obesity is associated with hyperlipidemia and nonalcoholic fatty liver disease (NAFLD). Fatty acids secreted from adipose tissue into the blood act as the source of lipid synthesis in the liver [27,28]. In our study, MetA100 reduced the serum NEFA, T-CHO, and LDL-CHO levels, and MetA50 increased the serum HDL-CHO levels while decreasing serum LDL-CHO levels. Consistent with the previous study, LV and HS also regulated serum lipid profiles [14,15]. In addition, the livers of MetA-treated mice were lighter, and triglyceride content was lower than that of the other groups. Histological data of the liver exhibited reduced numbers, and smaller sizes of hepatic lipid droplets in the MetA100-treated group, supporting our present data on corresponding the serum and liver lipid profiles. These results indicate that MetA may ameliorate fatty liver and hyperlipidemia, which may be accompanied by improved obesity. Next, we determined whether MetA would activate AMPK as another mechanism of these effects. AMPK plays an important role in lipid metabolism by stimulating fatty-acid oxidation and inhibiting lipogenesis [29,30]. Consistent with previous reports [9,16], we found that the MetA100 and its component LV and HS increased ACC. Regarding AMPK phosphorylation in the liver, we found that Met and LV, but not HS, increased the expression levels, especially MetA100, which showed more effectiveness in AMPK activation, at values that were even higher than those in the NFD group were. Moreover, MetA decreased expression levels of lipogenesis-related genes, SREBP1c and FAS, but increased the expression levels of fatty acid oxidation-related genes, PPARα and ACS1. This result indicates that AMPK activation may be responsible for the amelioration of adiposity, dyslipidemia, and glucose tolerance as well as fatty liver in the MetA group.

Next, we determined whether MetA could also improve glucose metabolism and insulin resistance in the HFD-treated mice. We found that MetA100 decreased the serum glucose levels and inhibited glucose elevation in IGTT. Furthermore, MetA increased the serum adiponectin levels and mRNA levels, but also lowered proinflammatory cytokines TNF-α and IL-6 levels. Adipocytes are hypertrophic in obese state, leading to low-grade chronic inflammation through molecular and cellular changes including increased proinflammatory cytokines in the WAT, which links obesity to the pathogenesis of insulin resistance and diabetes [31]. TNF-α and IL-6 disrupt insulin signaling by targeting the downstream proteins of insulin signaling pathways such as glucose transporter 4 and insulin receptor substrate-1 phosphorylation [32,33]. Adiponectin regulates glucose and lipid metabolism by AMPK activation [34,35]. In this regard, LV, HS, and MetA may improve the dysregulation of adipokine expression. Consequently, their beneficial effects on adipokines regulation might contribute to improving glucose metabolism and insulin resistance.

Our HPLC analysis revealed that MetA contained marker compounds, delphinidin-3-o-sambubioside, cyanidin-3-o-sambubioside, verbascoside, and isoverbascoside, consistent with previous studies that verbascoside and isoverbascoside, and delphinidin-3-o-sambubioside, cyanidin-3-o-sambubioside are found most abundantly in LV and HS, respectively [36,37]. Verbascoside, a phenylpropanoid glycosides, is known to possess multiple biological activities including antioxidant, anti-inflammatory, and anticancer as well as antiobesity effects with AMPK activation [9,38,39]. In particular, verbascoside decreases oxidative parameters as well as increases antioxidant activity and vitamin A and E levels. It also activates sirtuin isoform 1 (SIRT1) activity, which can regulate antioxidant genes and is an important regulator of many signaling pathways, such as AMPK activation, associated with obesity and metabolic disorders [40]. This suggests that the antioxidant effect of verbascoside might be regulated through the increase in SIRT1 activity, leading to treatment of obesity and related metabolic disorders through AMPK activation. Delphinidin-3-o-sambubioside and cyanidin-3-o-sambubioside are one the anthocyanins. There are many reports that anthocyanins have antioxidant, anti-inflammatory, and antiobesity effects by regulating glucose and lipid metabolism [17]. However, there is no evidence for glucose and lipid metabolism of delphinidin-3-o-sambubioside and cyanidin-3-o-sambubioside. These findings from previous reports suggest that these compounds could regulate obesity and its complications, and may contribute to antiobesity effects as bioactive compounds.

There are pharmacologic and nonpharmacologic strategies for controlling obesity. The nonpharmacological interventions include caloric restrictions (CR). CR reduced weight and leptin levels, but increased adiponectin levels, which were similar to the results of the present study. Despite the benefits of CR with respect to controlling obesity, long-term CR in humans is not feasible [41]. Accordingly, there has been an increasing interest in identifying compounds, such as phytochemicals that have an ability to act as CR mimetics. However, although phytochemicals and bioactive food compounds have beneficial effects against a broad range of pathologies, including obesity and related disorders, their use is limited owing to their low bioavailability due to high metabolism in the gastrointestinal tract. Similar to that of other phytochemicals, oral bioavailability of verbascoside from lemon verbena and cyanidin-3-o-sambubioside and delphinidin-3-o-sambubioside from hibiscus flower was low, although they have been reported to be rapidly absorbed from the gastrointestinal tract and then detected in rat plasma [42,43], although the pharmacokinetics of MetA in humans need to be analyzed. Interestingly, Boix-Castejon et al. studied MetA, an optimized combination of *Hibiscus sabdariffa* and lemon verbena (*Lippia citriodora*) polyphenols, which reduced body fat, blood pressure, and heart rate, and led to a more positive perception with respect to overall health status of overweight subjects compared with the placebo group. Additionally, it regulated satiety and hunger-related hormones: increased anorexigenic hormone levels, and decreased orexigenic hormone and leptin levels [44]. No adverse side effects of dietary supplementation of MetA were reported in this study [44]. Taken together, *H. sabdariffa* preparations, particularly *H. sabdariffa* tea and aqueous extracts, and lemon verbena did not show side effects in animals and humans [45,46]. Therefore, MetA could improve obesity and is considered safe, avoiding CR and low bioavailability limitations.

## 5. Conclusions

We found that MetA, a combination of LV and HS, reduced body-weight gain, WAT and liver weight, serum and hepatic lipid profiles, and serum glucose levels. Furthermore, MetA improved glucose tolerance and cold-induced thermogenesis, increased the adiponectin serum and gene-expression levels, while decreasing the leptin serum and gene and proinflammatory cytokines, TNF-α, and IL-6 gene-expression levels. In addition, MetA decreased the expression of the adipogenesis-related genes, C/EBPα, PPARγ, SREBP1c, and FAS, while increasing that of thermogenesis-related genes, UCP1 and UCP2, in the WAT, but also, it increased phosphorylation of AMPK and fatty oxidation-related genes, PPARα and ACS1, while decreasing the lipogenesis-related genes SREBP1c and FAS in the liver of HFD-induced obese mice. The results observed with MetA were more significant than that of with HB and LV alone, indicating the synergistic effect of the two extracts. In conclusion, MetA synergistically ameliorated adiposity, hyperlipidemia, and glucose tolerance in HFD-induced obese mice, which was accompanied by decreased adipogenesis, and increased thermogenesis and AMPK activation. Our results provide novel and critical evidence to support the use of MetA in the management of obesity and metabolic diseases.

## Figures and Tables

**Table 1:** Oligonucleotide and probe sequences.

Genes	GeneBank Accession NO	Sequences
C/EBPα	BC011118.1	Forward:5′-TGGACAAGAACAGCAACGAGTAC-3′ Reverse: 5′-CGGTCATTGTCACTGGTCAACT-3′
PPARγ	NM_011146	FAM: 5′-TCGGAATCAGCTCTGTGGACCTCTCC-3′
SREBP1c	BC056922.1	Forward: 5′-AGCCTGGCCATCTGTGAGAA-3′ Reverse: 5′-CAGACTGGTACGGGCCACAA-3′;
aP2/FABP4	NM_024406	Forward: 5′-TGGGAACCTGGAAGCTTGTCTC-3′ Reverse: 5′-GAATTCCACGCCCAGTTTGA-3′
FAS	NM_007988	Forward: 5′-CTGAGATCCCAGCACTTCTTGA-3′ Reverse: 5′-GCCTCCGAAGCCAAATGAG-3′;
SCD1	AH002082.2	Forward: 5′-CATCGC CTGCTCTACCCTTT-3′ Reverse: 5′-GAACTG CGCTTGGAAACCTG-3′
DGAT1	NM_10046	Forward: 5′-TGCTACGACGAGTTCTTGAG-3′ Reverse: 5′-CTCTGCCACAGCATTGAGAC-3′
PPARα	BC016892	Forward: 5′-GCCTGTCTGTCGGGATGT-3′ Reverse: 5′-GGCTTCGTGGATTCTCTTG-3′
CTP1b	AF017174.2	Forward: 5′-GTCGCTTCTTCAAGGTCTGG-3′ Reverse: 5′-AAGAAAGCAGCACGTTCGAT-3′
ACS1	U15977.1	Forward: 5′-TCCTACAAAGAGGTGGCAGAACT-3′ Reverse: 5′-GGCTTGAACCCCTTCTGGAT-3′
ACOX	BC056448	Forward: 5′-CAGGAAGAGCAAGGAAGTGG-3′ Reverse: 5′-CCTTTCTGGCTGATCCCATA-3′
UCP1	NM_009463.3	Forward: 5′-GACTCAGTCCAAGAGTACTTCTCTTC -3′ Reverse: 5′-GCCGGCTGAGATCTTGTTTC-3′
UCP2	NM_011671	Forward: 5′-CCGCATTGGCCTCTACGACTCT-3′ Reverse: 5′-CCCCGAAGGCAGAAGTGAAGTG-3′
Adiponectin	NM_009605	Forward: 5′-GTCTCAGCTGTCGGTCTTCCCCT-3′ Reverse: 5′-CCCTGGCTTTATGCTCTTTGC-3′
Leptin	NM_008493	Forward: 5′-CCAAAACCCTCATCAAGACC-3′ Reverse: 5′-GTCCAACTGTTGAAGAATGTCCC-3′
GAPDH	NM_008084	VIC: 5′-TGCATCCTGCACCACCAACTGCTTAG-3′

**Table 2:** Effect of LV, HS, and the MetA-mixed extract of LV and HS on biochemistry parameters in serum and liver tissue of HFD-induced obese mice.

	NFD	HFD	HFD				
			GC_245	LV_100	HS_100	MetA_50	MetA_100
TG (mg/dL)	91.5 ± 4.1 ^a^	121.9 ± 10.5	106.6 ± 6.0 ^ab^	75.7 ± 4.5 ^ac^	100.4 ± 3.0 ^ab^	109.4 ± 8.0 ^ab^	105.4 ± 2.5 ^ab^
NEFA (mEq/L)	3.0 ± 0.1 ^a^	3.3 ± 0.2 ^a^	2.8 ± 0.1 ^a^	2.5 ± 0.1 ^b^	2.6 ± 0.1 ^b^	3.3 ± 0.17 ^a^	2.6 ± 0.1 ^b^
T-CHO (mg/dL)	118.4 ± 2.4 ^a^	317.1 ± 28.3 ^b^	186.8 ± 10.4	262.8 ± 36.2 ^b^	321.4 ± 34.4 ^b^	282.2 ± 15.7 ^b^	149.7 ± 6.3
LDL-CHO (mg/dL)	10.1 ± 0.5 ^a^	16.7 ± 1.5 ^b^	8.9 ± 0.8 ^ac^	13.2 ± 1.7 ^ab^	13.3 ± 1.4 ^ab^	11.3 ± 0.6	10.5 ± 0.6 ^ac^
HDL-CHO (mg/dL)	48.4 ± 1.1 ^a^	52.9 ± 2.2 ^a^	54.6 ± 2.6 ^a^	59.3 ± 3.4 ^a^	60.1 ± 2.6 ^b^	72.0 ± 3.6 ^c^	59.3 ± 2.5 ^a^
ALT (mg/dL)	20.7 ± 0.7 ^a^	61.6 ± 12.4 ^b^	24.8 ± 2.2 ^a^	21.4 ± 1.3	35.8 ± 5.7 ^a^	37.5 ± 8.3 ^a^	29.2 ± 4.1 ^a^
AST (mg/dL)	81.1 ± 8.6	118.0 ± 35.1	77.9 ± 9.6	91.4 ± 16.3	80.0 ± 7.1	82.1 ± 5.0	81.1 ± 5.8
Creatinine (mg/dL)	0.29 ± 0.01	0.34 ± 0.01	0.35 ± 0.01	0.33 ± 0.02	0.29 ± 0.01	0.34 ± 0.02	0.35 ± 0.01

TG, Triglycerides; NEFA, nonesterified fatty acid; T-CHO, Total cholesterol; HDL-CHO, high-density lipoprotein-CHO; LDL-CHO, low-density lipoprotein-CHO; AST, aspartate aminotransferase; ALT, alanine aminotransferase; HFD, 60% high-fat-diet control group; HFD + GC_245, HFD contains *Garcinia cambogia* extract (245 mg/kg)-fed group; HFD + LV_100, HFD contains lemon verbena extract (100 mg/kg)-fed group; HFD + HS_100, HFD contains *Hibiscus* flower extract (100 mg/kg)-fed group; HFD + MetA_50, HFD contains the MetA-mixed extract of lemon verbena plus *Hibiscus* flower (50 mg/kg)-fed group; HFD + MetA_100, HFD contains the MetA-mixed extract of lemon verbena plus *Hibiscus* flower (100 mg/kg)-fed group. Values are expressed as means ± SEM (*n* = 10). ^a^^–c^ Values within arrow with different letters are significantly different from each other at *p* < 0.05 as determined by Turkey’s multiple-range test.
